# Purifying Gold Scrap

**Published:** 1906-04

**Authors:** E. F. Evans


### PURIFYING GOLD SCRAP.

If all dentists would practice the United States mint method they would not have to sell gold scraps at a loss.

Have a bottle of nitric acid and another of hydro-chloric acid and mix when used. To one part nitric acid add from two to two and one-half to three parts hydro-chloric acid. Then add gold scraps of all carats. After gold is dissolved pour off gold acid from the white silver powder deposit, then thoroughly dilute the gold acid with water, which should turn an orange color. Make a saturated solution of green copperas and add to the diluted gold acid, which should turn a dark brown muddy color. Set aside until clear and the gold will be deposited as a brown powder; pour off liquid and wash gold several times in hot water, then pour sulphuric acid over gold and let stand for a few hours, which will dissolve any iron wire etc.; then wash and place gold powder and flux on depression in flat piece of charcoal and cover with another' piece, then melt with blow pipe. The gold will be pure, unless scraps from gold money is used, then the copper will remain, which a dentist has not heat enough at his command to remove. The gold sold at the depots contains silver, and, treated by this method, the result will be pure gold. Then reduce with silver to carat wanted. The gold acid will stain the fingers, so you should wear gloves at this work.—*Dr. E. F. Evans, Texas Dental Journal.*

